# Short‐Term Outcomes and Cost Drivers of Emergency Surgery for Acute Abdominal Disease in Super‐Elderly Patients: A Study in the Japanese Tertiary Care Hospital

**DOI:** 10.1002/ags3.70222

**Published:** 2026-04-10

**Authors:** Yuta Kobayashi, Ryo Oikawa, Yumi Shibuya, Masakazu Tatsuno, Atsuya Kamiyama, Tsuyoshi Ozawa, Keiichiro Hara, Shojiro Hata, Hirokazu Yamaguchi

**Affiliations:** ^1^ Department of Gastroenterological Surgery Showa General Hospital Kodaira Japan

## Abstract

**Background:**

Although emergency surgery for acute abdominal disease in super‐elderly patients is often associated with high risk and substantial resource utilization, the relationship between short‐term outcomes and inpatient medical costs remains unclear.

**Methods:**

In total, 247 patients aged ≥ 85 years who underwent emergency surgery for acute abdominal disease were analyzed. Inpatient costs were assessed using total inpatient claim points and the fee‐for‐service component under Japan's diagnosis‐based payment system. Multivariable analyses were performed to identify risk factors for postoperative complications, non‐home discharge, and excess inpatient costs.

**Results:**

Overall complications occurred in 44.1% of patients, major complications in 19.4%, and 72.1% were discharged home. Mortality was predominantly observed in strangulated bowel obstruction, bowel perforation, and gastrointestinal ischemia or bleeding (8.1%). Multivariable analyses identified American Society of Anesthesiologists physical status (odds ratio [OR] 3.52, *p* < 0.001) and operative time (OR 2.10, *p* < 0.001) as independent risk factors for major complications, while age (OR 1.17, *p* < 0.001), dementia (OR 2.34, *p* = 0.006), and major complications (OR 2.87, *p* = 0.005) were associated with non‐home discharge. Total inpatient claim points were nearly doubled in patients with major complications (*p* < 0.001), and residual cost analysis demonstrated disproportionately high inpatient costs among patients without major complications, independent of length of stay.

**Conclusions:**

Emergency abdominal surgery can be performed with acceptable short‐term outcomes and high likelihood of discharge to home in carefully selected super‐elderly patients. Inpatient costs among patients without major complications might be modifiable through optimization of perioperative management and resource utilization.

## Graphical abstract

This retrospective study analyzed patients aged ≥ 85 years undergoing emergency abdominal surgery, focusing on short‐term outcomes and inpatient cost structure under the Japanese DPC system. Although major complications occurred in 19.4% of patients, more than 70% were discharged home. While major complications were associated with unavoidable increases in medical costs, residual cost analysis revealed disproportionately high inpatient costs even among patients without major complications, which might be modifiable through optimization of perioperative management and resource utilization.

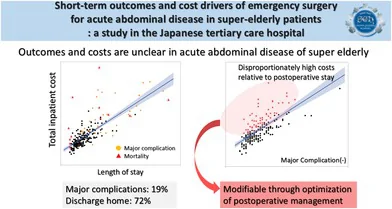

## 1 Introduction

Traditionally, emergency surgery for acute abdominal diseases in super‐elderly patients aged 85 years and older has been regarded as high risk, largely because this population frequently presents with multiple comorbidities [1, 2]. Previous clinical consensus has indicated that such patients are more susceptible to postoperative complications, and that declines in activities of daily living and cognitive function often lead to prolonged hospitalization and increased medical costs [3]. However, with the extension of healthy life expectancy, the number of super‐elderly patients who maintain good functional status and independence in daily activities has been increasing. Some researchers have recently reported that aggressive surgery can be performed safely even in these aged patients, with successful discharge to their own homes [4]. In this context, conventional perioperative risk stratification and cost evaluation frameworks may no longer be appropriate and therefore warrant reappraisal [5, 6].

From an ethical standpoint, the initial approach to even super‐elderly, high‐risk patients should not be unduly restricted, and the best possible treatment should be offered. At the same time, given the constraints of limited healthcare resources and public funding, the indiscriminate use of high‐cost medical care is not sustainable, and the delivery of efficient, value‐based care is increasingly required [7]. In real‐world clinical practice, some patients incur unnecessarily prolonged hospital stays and increased medical costs despite the absence of significant postoperative complications, often due to non‐medical factors. Optimization of healthcare resource utilization therefore remains a critical and unresolved issue.

This study uses real‐world data from a regional tertiary care hospital to clarify the surgical management and short‐term outcomes of super‐elderly patients undergoing emergency surgery for acute abdominal disease. Furthermore, we aimed to identify clinical risk factors and to examine which patient subgroups and cost components contribute disproportionately to inpatient medical expenditures, thereby exploring potential targets for improving the efficiency of perioperative care without compromising clinical outcomes.

## 2 Methods

### 2.1 Study Population

The clinical records of 2691 consecutive patients admitted for acute abdominal disease from January 2016 to 2024 at Showa General Hospital were reviewed. Among them, 248 patients aged ≥ 85 years who underwent emergency surgery were identified. After excluding one patient who underwent re‐operation associated with elective surgery, a total of 247 patients were included. 220 patients underwent emergency surgery within 48 h of presentation and 27 patients required unplanned emergency surgery due to clinical deterioration during conservative management (Figure S1).

This study was conducted in accordance with the Declaration of Helsinki and the ethical guidelines for clinical study in Japan under approval of the Institutional Review Board at Showa General Hospital (No. 395).

### 2.2 Indication of Emergency Surgery and Postoperative Management for the Super‐Elderly Patients

Upon surgical consultation for a patient with acute abdominal disease, disease severity was assessed based on vital signs, physical examination findings, laboratory tests, and contrast‐enhanced computed tomography imaging whenever not contraindicated. Patients presenting with uncontrollable hemodynamic instability, multiple organ failure, or respiratory failure, as well as those with a history of severe cardiac, pulmonary, or neurological disease, were generally considered unsuitable for surgical intervention. Surgical indication was not determined by age; rather, preexisting comorbidities, functional status, and American Society of Anesthesiologists physical status (ASA‐PS) were comprehensively evaluated, and surgical tolerance was discussed in detail with anesthesiologists. For patients with dementia who lacked decisional capacity, surgery was sometimes performed based solely on family preference alone, after thorough explanation of the expected benefits and risks.

Patients with gastrointestinal perforation presenting with diffuse peritonitis were, in principle, considered surgical candidates. Upper gastrointestinal perforations were treated by closure of the perforation with omental patch repair, followed by irrigation and drainage. Lower gastrointestinal perforations were managed primarily with stoma creation and lavage; however, in cases of right‐sided colonic perforation with localized contamination, primary anastomosis was occasionally performed. Appendicitis was generally treated surgically, although percutaneous drainage was performed in selected cases of perforated appendicitis with localized peritonitis. Mild appendicitis was treated conservatively with antibiotics. For acute cholecystitis, early surgery or percutaneous transhepatic gallbladder drainage was selected according to the Tokyo Guidelines 2018 [8]. Patients with strangulated bowel obstruction were managed surgically as a general rule. Irreversible small bowel ischemia was treated with one‐stage resection and anastomosis. For mechanical bowel obstruction, conservative therapy with nasogastric decompression was attempted initially, and surgery was performed if the response was inadequate. The choice between laparoscopic and open surgery depended on institutional resources and the patient's history of abdominal surgery.

Postoperative management in the intensive care unit (ICU) was provided when required based on the patient's overall condition. Early postoperative management focused on stabilization of hemodynamics and fluid balance, with close attention to infection control. Nutritional support and rehabilitation were provided in accordance with the Enhanced Recovery After Surgery (ERAS) protocol, with the goal of enabling patients to return home whenever feasible.

### 2.3 Evaluation of Surgical Outcomes

The patient's conditions were categorized into eight major groups. Because mortality was concentrated in the first four categories, they were classified as Group A: strangulated bowel obstruction, upper gastrointestinal perforation, lower gastrointestinal perforation, and gastrointestinal ischemia or bleeding. The remaining conditions were classified as Group B: appendicitis, cholecystitis, mechanical bowel obstruction, and others. The others included Fournier's gangrene, traumatic splenic injury, and common bile duct stone and pneumatosis intestinalis. Postoperative outcomes were evaluated using the Clavien–Dindo (CD) classification [9], with major complications defined as CD Grade 3 or higher and deaths within 90 days classified as Grade 5. Multivariable analyses were performed to identify the risk of postoperative complications and non‐home discharge.

### 2.4 Evaluation of Financial Outcomes

In Japan, inpatient reimbursement under the Diagnosis Procedure Combination (DPC) system consists of a fixed per‐diem bundled payment and an additional fee‐for‐service component for services not covered by the bundle [10]. All cases were managed under the DPC reimbursement system, and no additional claims outside the DPC framework were made. DPC coding was performed according to standardized institutional rules by trained medical coders based on physician documentation. Coding accuracy was routinely reviewed through internal audits to ensure consistency.

No changes in coding policy occurred during the study period. For each patient, we extracted total inpatient claim points, representing the overall reimbursed cost for the hospitalization, and the fee‐for‐service component, defined as the sum of itemized points billed outside the DPC bundled payment. This fee‐for‐service component largely reflects postoperative resource use, including rehabilitation, additional procedures, imaging, laboratory tests, and other non‐bundled services. To further characterize the financial structure, total inpatient claim points were decomposed into five components: (1) total inpatient claim points, (2) surgical procedure points, (3) anesthesia and operating room consumables, (4) DPC bundled payment points, and (5) fee‐for‐service points. By definition, the total inpatient claim points represent the sum of these components (1 = 2 + 3 + 4 + 5), allowing evaluation of the relative contribution of each cost domain to overall hospitalization expenditure. All surgical procedures in this cohort were performed under the national health insurance system and were fully covered within the scope of public insurance. Because all patients were ≥ 85 years old and therefore classified as late‐elderly beneficiaries under the Japanese insurance system, their individual copayment was uniformly 10%. One reimbursement point corresponds to 10 Japanese yen.

### 2.5 Analysis of Inpatient Costs and Residual Evaluation

Inpatient medical costs were evaluated using total inpatient claim points and the fee‐for‐service component, as defined above. Postoperative length of stay (LOS) was treated as a continuous explanatory variable reflecting the duration of inpatient care after surgery. First, linear regression analyses were performed in the entire cohort to examine the relationship between LOS and each cost variable. Scatter plots with fitted regression lines and 95% confidence intervals were generated to visualize cost distributions. To further explore disproportionately high inpatient costs not explained by postoperative stay, additional analyses were conducted in patients without major postoperative complications (Clavien–Dindo grade ≤ 2). In this subgroup, separate linear regression models were constructed with LOS as the independent variable and with total inpatient claim points and the fee‐for‐service component as the dependent variables. For each model, regression residuals were calculated as the difference between observed and expected claim points based on postoperative LOS. Residual plots were used to assess the distribution of costs relative to LOS and to identify cases with disproportionately high costs beyond what would be expected from prolonged hospitalization alone.

Based on these residuals, patients in the upper quartile of cost residuals were defined as having excess inpatient costs, and this definition was subsequently used for risk factor analyses.

### 2.6 Statistical Analysis

Continuous values are expressed as the median values (range) and compared using Wilcoxon's rank‐sum test. Categorical variables are expressed as numbers (%) and compared using Fisher's exact test or the chi‐squared test, as appropriate. *p* < 0.05 was considered as indicative of statistical significance. Additionally, to identify potential risks associated postoperative complications, non‐home discharge and high medical costs, multivariable logistic regression with backward elimination was performed. To avoid overfitting, only factors that showed a statistically significant association with postoperative course at *p* < 0.1 were included in the final model. All the statistical analyses were conducted using the JMP software (version14; SAS Institute Inc., Cary, NC) or IBM SPSS software (Ver. 26.0; SPSS Inc., IL, USA).

## 3 Results

### 3.1 Patient Characteristics and Overall Outcomes

Baseline characteristics and postoperative outcomes of 247 patients in the study are summarized in Table 1. Among the cohort, 44.9% had an ASA‐PS of ≥ 3, 35.6% were receiving anticoagulant therapy, and 34.4% had dementia; 82.0% were admitted from home. Strangulated bowel obstruction accounted for 36.8% of cases, followed by acute cholecystitis (19.4%) and mechanical bowel obstruction (14.2%). Overall postoperative complications occurred in 44.1%, and major complications in 19.4%. A total of 72.1% were discharged home.

**TABLE 1:** Base line characteristics and postoperative outcomes.

Number of patients	247
Age, year	87 (85–99)
Sex ratio (male/female)	121/126
BMI, kg/m^2^	20.2 (13.3–33.8)
ASA‐PS score 1/2/3/4/5	1 (0.4)/135 (54.7)/88 (35.6)/21 (8.5)/2 (0.8)
Comorbidities or past disease	
Ischemic heart disease	31 (12.6)
Other cardiovascular disease	172 (69.6)
Diabetes	46 (18.6)
Chronic kidney disease	26 (10.5)
Respiratory disease	32 (13.0)
Central nerve system disease	49 (19.8)
Use of anticoagulant/antiplatelet drug	88 (35.6)
Use of steroid	10 (4.0)
CCI	1 (0–8)
Dementia	85 (34.4)
Place of residence before admission	
Home/nursing facility/hospital	200 (82.0)/33 (13.5)/11 (4.5)
No support required/unknown	115 (46.6)/29 (11.7)
Support levels (requiring partial assistance)^a^ 1/2	16 (6.5)/17 (6.9)
Care levels (requiring daily caregiving)^b^ 1/2/3/4/5	29 (11.7)/34 (13.8)/10 (4.0)/15 (6.1)/2 (0.8)
Group A disease	136 (55.1)
Strangulated bowel obstruction	91 (36.8)
Upper bowel perforation	10 (4.0)
Lower bowel perforation	26 (10.5)
Gastrointestinal ischemia or bleeding	9 (3.6)
Group B disease	111 (44.9)
Acute appendicitis	24 (9.7)
Acute cholecystitis	48 (19.4)
Mechanical bowel obstruction	35 (14.2)
Others^c^	4 (1.6)
Preoperative systemic condition	
SIRS score	1 (0–4)
qSOFA score	0 (0–3)
Postoperative outcomes	
Overall complication	109 (44.1)
Minor complication	61 (24.7)
Major complication	48 (19.4)
Postoperative length of stay, days	11 (1–76)
Discharge to home	178 (72.1)

Abbreviations: ASA‐PS, American association of anesthesia‐physical status; BMI, body mass index; CCI, Charlson comorbidity index; qSOFA, Quick Sequential Organ Failure Assessment; SIRS, systemic inflammatory response syndrome.

^a^ Support level (Yō‐shien).

^b^ Care level (Yō‐kaigo).

^c^ Fournier's gangrene, traumatic splenic injury, common bile duct stone.

### 3.2 Risk Factors for Postoperative Outcomes

Table 2 summarizes perioperative outcomes stratified by the presence or absence of major complications. Major complications occurred across disease categories and were associated with prolonged LOS, an increased rate of non‐home discharge, and higher inpatient costs. Multivariable analyses identifying risk factors for overall complication, major complication, and non‐home discharge are summarized in Table 3. Higher ASA‐PS and longer operative time were significant risk factors for overall complications (OR 2.17, *p* < 0.001 and OR 1.90, *p* < 0.001, respectively) and major complication (OR 3.52, *p* < 0.001 and OR 2.10, p < 0.001 respectively). Group A disease was also associated with overall complication (OR 2.04, *p* = 0.012). Higher age, dementia and major complications were the significant predictors for non‐home discharge (OR 1.17, *p* = 0.001, OR 2.34, *p* = 0.006 and OR 2.87, *p* = 0.005, respectively).

**TABLE 2:** Perioperative outcomes and financial outcomes.

	C‐D ≤ 2 (*N* = 199)	C‐D ≥ 3 (*N* = 48)	*p*
Disease group A^a^/B^b^	106 (53.3)/93 (46.7)	30 (62.5)/18 (37.5)	0.263
Age	87 (85–99)	88 (85–97)	0.812
Sex	104 (52.3)/95 (47.7)	22 (45.8)/26 (54.2)	0.520
ASA‐PS (1/2/3/4/5)	1/122/66/10/0	0/13/22/11/2	< 0.001
BMI, kg/m^2^	20.0 (13.3–33.8)	20.8 (15.6–29.1)	0.110
SIRS	1 (0–4)	1 (0–4)	0.001
Laparoscopic procedure	16 (8.0)	2 (4.2)	0.538
Operation time, min	97 (15–251)	131 (41–335)	< 0.001
Estimated blood loss, mL	30 (0–4710)	60 (0–4195)	0.016
Transfusion	11 (5.5)	10 (20.8)	0.002
ICU admission	0	22 (45.8)	< 0.001
Postoperative length of stay, days	10 (2–51)	25 (1–76)	< 0.001
Nonhome discharge	46 (23.1)	23 (47.9)	0.001
Total inpatient claim points	115 084 (24466–315 106)	273 646 (70685–610 964)	< 0.001
Fee‐for‐service component	20 782 (1511–103 667)	67 251 (2607–248 752)	< 0.001

Abbreviations: ASA‐PS, American Society of Anesthesiologists physical status; ICU, intensive care unit; SIRS, systemic inflammatory response syndrome.

^a^ Strangulated bowel obstruction, Upper/Lower bowel perforation and Gastrointestinal ischemia or bleeding.

^b^ Appendicitis, Cholecystitis, Mechanical bowel obstruction and others.

**TABLE 3:** Multivariate analysis of factors associated with postoperative outcomes.

	*p* ^a^	Odds ratio	95% CI
Overall complication			
ASA‐PS (per 1 point)	< 0.001	2.17	1.42–3.33
Group A disease^b^	0.012	2.04	1.17–3.58
Operation time (per 1 h)	< 0.001	1.90	1.38–2.61
Major complication			
ASA‐PS (per 1 point)	< 0.001	3.52	2.11–5.89
Operation time (per 1 h)	< 0.001	2.10	1.44–3.08
Non‐home discharge			
Age (per 1 year)	0.001	1.17	1.06–1.28
Dementia	0.006	2.34	1.27–4.33
Major complication	0.005	2.87	1.37–6.03

*Note:* Multivariate logistic regression was applied with stepwise backward selection. Initially, all factors presenting with *p* < 0.5 in the univariate analysis were included in the model. Then factors that showed no or limited statistically significant association (*p* > 0.1) with overall complication, major complication and non‐home discharge adjusted for the remaining factors in the model were deleted from the model in stepwise fashion. The 10 tested variables for overall and major complication were as follows: age, sex (male), Group A disease, dementia, ASA‐PS, SIRS, operation time, blood lost, laparoscopic procedure, a need for daily care or support. The 11 tested variables for nonhome discharge were the 10 variables above listed and major complication.

Abbreviations: 95% CI, 95% confidence interval; ASA‐PS, American association of anesthesia‐physical status; SIRS, systemic inflammatory response syndrome.

^a^ Based on likelihood test adjusted for the other factors in the final model.

^b^ Strangulated bowel obstruction, Upper/Lower bowel perforation and Gastrointestinal ischemia or bleeding.

### 3.3 Subgroup Analyses by Surgical Invasiveness and Disease Condition

Comparisons of baseline characteristics and short‐term postoperative outcomes between Group A and Group B are shown in Table S1A. Patients in Group A had higher systemic inflammatory response syndrome (SIRS) scores (*p* = 0.035), and were less likely to undergo laparoscopic surgery (2.9% vs. 12.6%, *p* = 0.006). Group A also had higher rates of ICU admission (13.2% vs. 3.6%, *p* = 0.012) and mortality (8.1% vs. 0.9%, *p* = 0.014). In contrast, there were no significant differences between the groups in total inpatient claim points or in the fee‐for‐service component (*p* = 0.071 and *p* = 0.236, respectively).

Comparisons according to bowel surgical procedure type are shown in Table S1B. Approximately 30% of bowel obstruction cases required bowel resection, and 81% of lower gastrointestinal perforations required stoma creation. The incidence of major complications, postoperative LOS, and treatment costs increased linearly across the three groups (no bowel resection, resection with anastomosis, and resection with stoma) (*p* < 0.05, respectively). In addition, a comparison between contaminated infectious conditions and intestinal ischemia or bleeding is presented in Table S1C. The contaminated infectious condition group showed significantly longer operative time, greater blood loss (*p* < 0.001, respectively), a higher rate of major complications (*p* = 0.024), and higher total inpatient costs (*p* = 0.037). The data of LOS and hospitalization costs associated with pneumonia, intra‐abdominal abscess, and anastomotic leakage are presented in Table S1D. Although the incidence was low, anastomotic leakage markedly increased both LOS and hospitalization costs.

### 3.4 Distribution of Total Inpatient Claim Points

**FIGURE 1:**
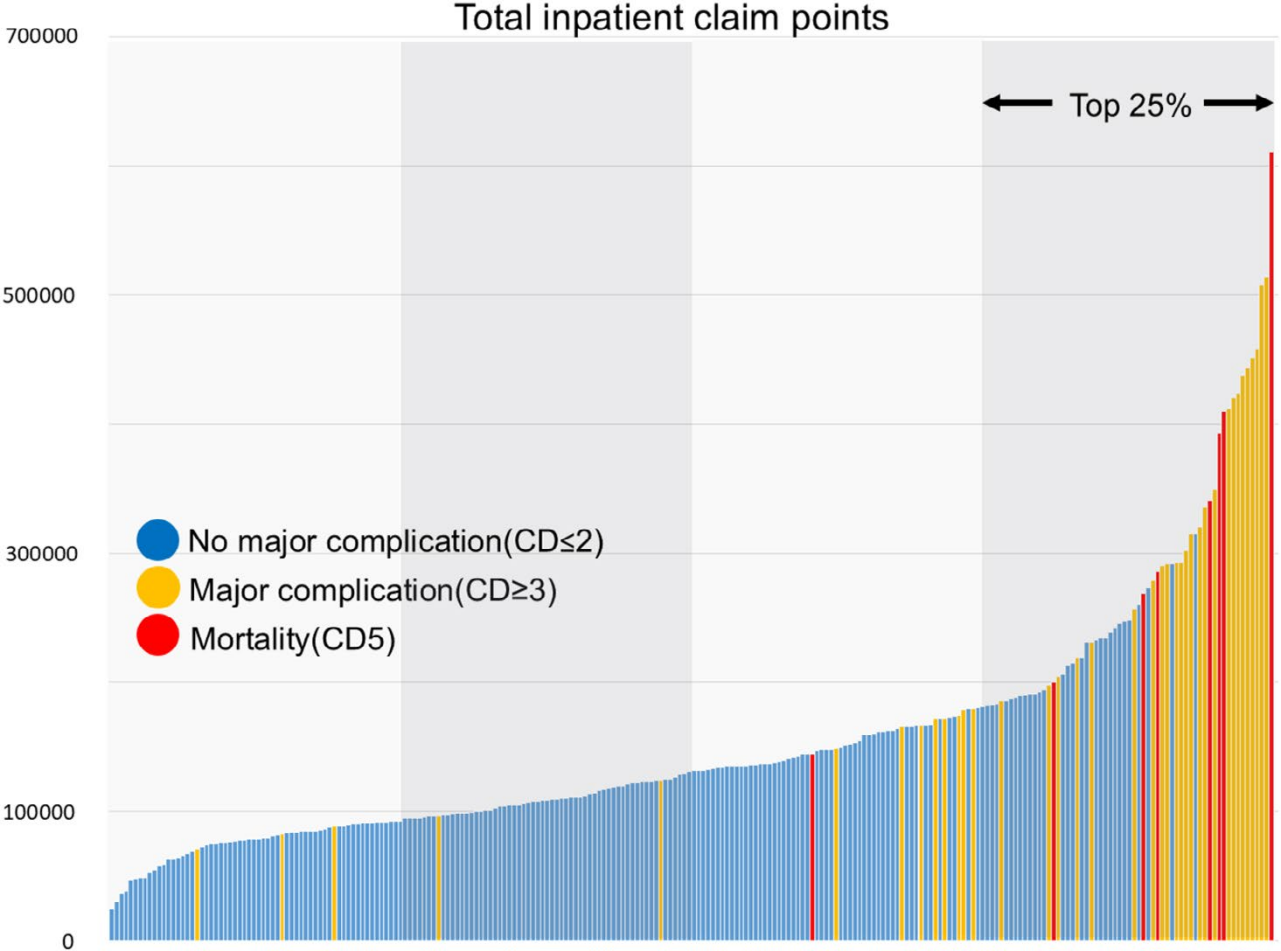
Distribution of total inpatient claim points. The shaded area indicates the upper quartile. Colors represent patients without major complications, with major complications, and mortality.

Figure 1 demonstrates a markedly skewed distribution of total inpatient claim points, with a steep increase observed in the upper quartile. Although major postoperative complications were concentrated in this high‐cost region, a subset of patients without major complications also exhibited disproportionately high costs.

### 3.5 Association Between Postoperative Length of Stay and Medical Costs

**FIGURE 2:**
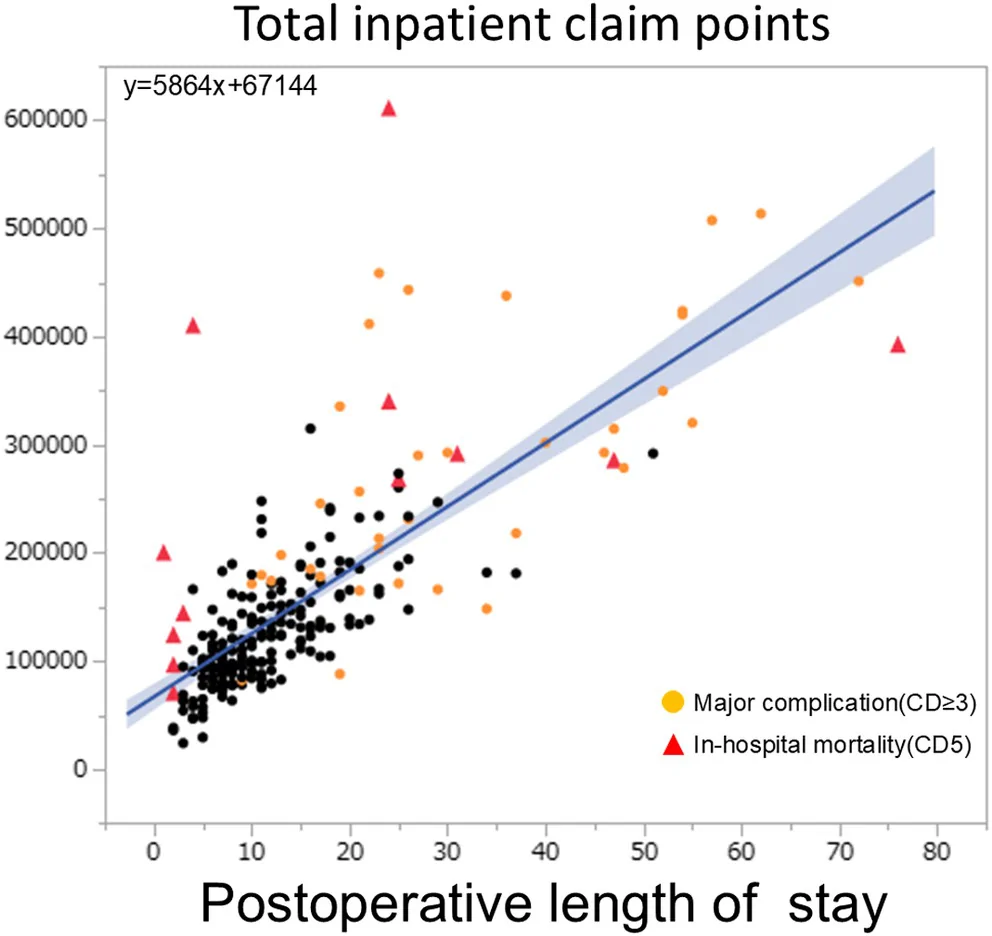
Relationship between postoperative length of stay and total inpatient claim points. Dots represent individual patients, with colors indicating mortality and major complications.

Figure 2 displays that total inpatient claim points were positively correlated with LOS. Higher costs were generally associated with prolonged hospitalization; however, substantial variability was noted, particularly among patients without major complications.

### 3.6 Residual Analysis in Patients Without Major Complications

**FIGURE 3:**
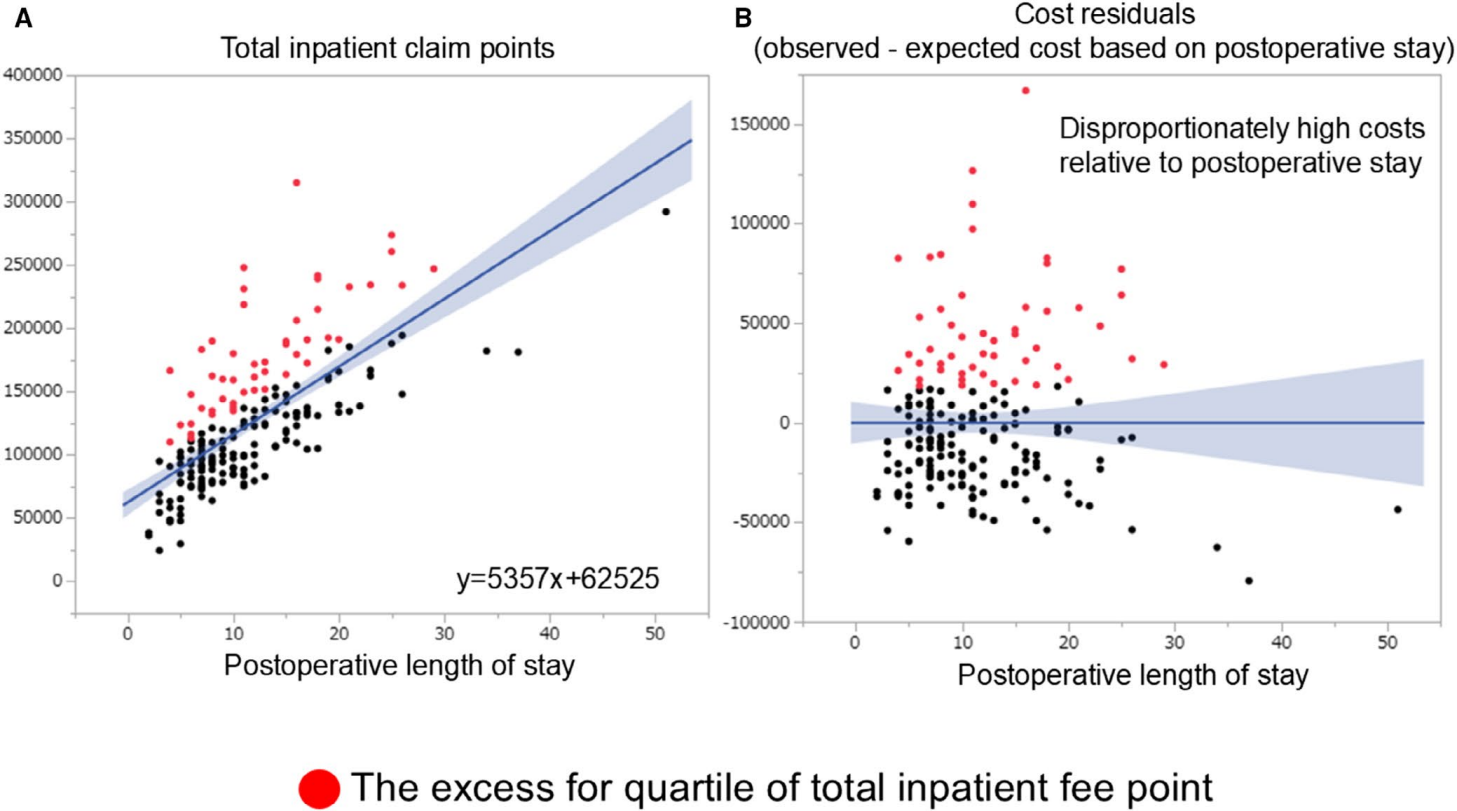
Association between postoperative length of stay and total inpatient claim points in patients without major complications. (A) Scatter plot with regression line. (B) Residual plot showing disproportionately high‐cost cases.

**FIGURE 4:**
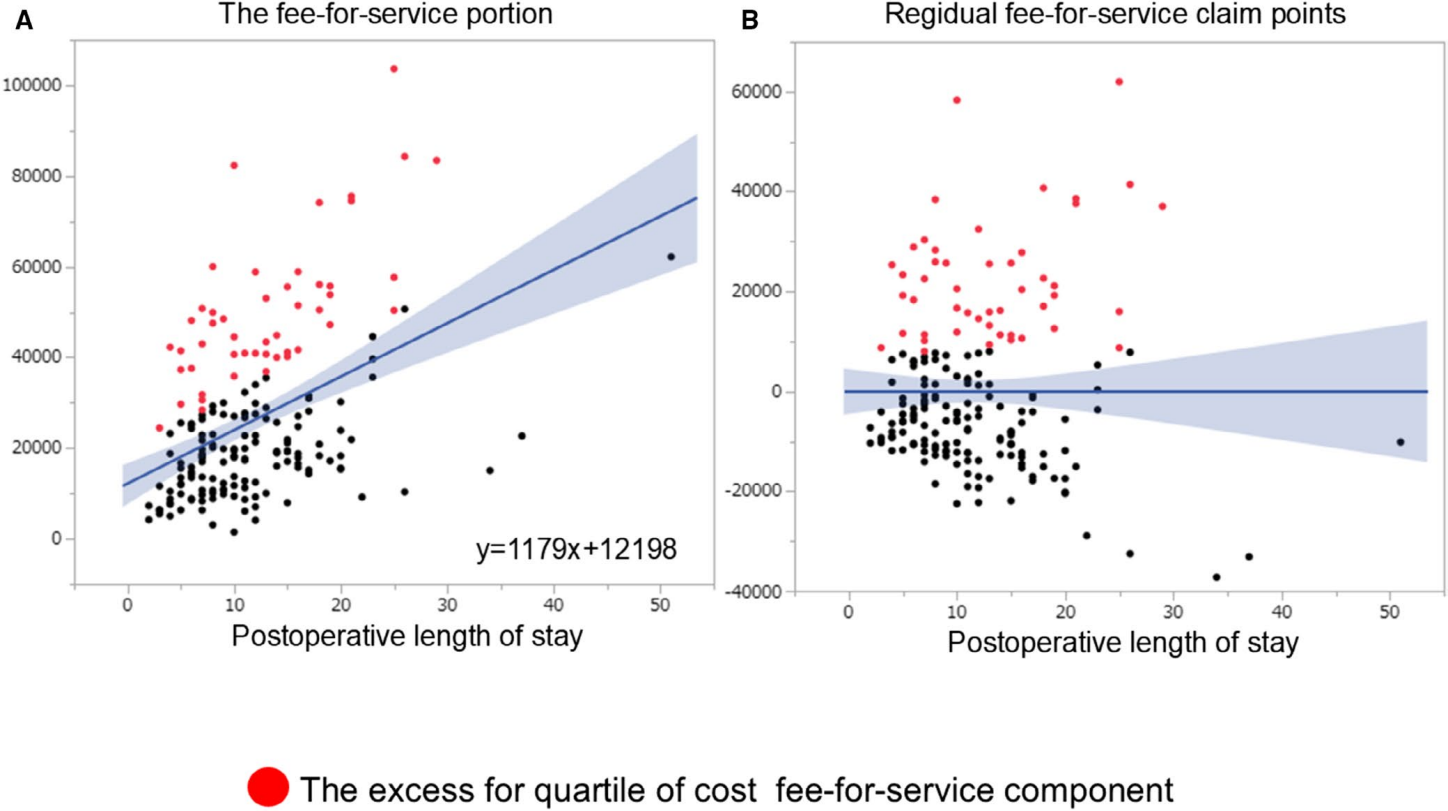
Association between postoperative length of stay and fee‐for‐service component in patients without major complications. (A) Scatter plot with regression line. (B) Residual plot showing disproportionately high‐cost cases.

To further explore potentially avoidable medical costs, residual analyses were performed among patients without major complications (*n* = 199). Linear regression models were constructed to evaluate the relationships between postoperative LOS and total inpatient claim points, as well as between LOS and the fee‐for‐service component. As shown in Figures 3 and 4, although LOS was positively associated with both cost measures, a subset of patients demonstrated disproportionately high residual costs relative to their LOS. These patients were predominantly concentrated in the upper quartile of both cost measures, suggesting excessive resource utilization not explained by clinical severity or postoperative complications. Cost components were compared between the excess and non‐excess groups (Table S2). The total fee‐for‐service points were significantly higher in the excess group. However, no significant differences were observed in surgical procedure points or anesthesia and operating room consumables between the two groups (*p* = 0.080, *p* = 0.187, respectively).

### 3.7 Risk Factors for Excessive Medical Costs Without Major Complications

Among patients without major complications, multivariable analyses were conducted to identify factors associated with excessive medical costs, defined as inclusion in the upper quartile of both cost measures (Table 4). Higher ASA‐PS score, higher SIRS score, Group A disease, and longer operative time were independently associated with excessive total inpatient claim points. Similar associations were observed when excessive costs were defined using the fee‐for‐service component, indicating that postoperative management and non‐procedural care contributed substantially to cost escalation even in the absence of major complications. In addition, using the median LOS as the cutoff, patients were divided into two groups: short stay group (LOS ≤ 10 days) and long stay group (LOS ≥ 11 days). Within each group, multivariable analyses were conducted to identify predictors of excess total inpatient claim points and excess fee‐for‐service points (Table S3). In the short‐stay group, sex, higher ASA‐PS, higher SIRS score, and longer operation time functioned as significant cost drivers. In contrast, in the long‐stay group, only operation time was identified as a significant cost driver.

**TABLE 4:** Multivariable analysis of factors associated with excess hospitalization cost in patients without major postoperative complications.

	*p* ^a^	Odds ratio	95% CI
The excess cost for quartile of total inpatient fee point (the top 25%)			
ASA‐PS	0.012	2.25	1.19–4.26
SIRS score	0.003	1.84	1.24–2.74
Group A disease^b^	0.045	2.38	1.02–5.58
Operation time (per 1 h)	< 0.001	3.71	2.20–6.26
The excess cost for quartile of fee‐for‐service component (the top 25%)			
Blood lost (per 100 mL)	0.025	1.18	1.02–1.36

*Note:* Multivariate logistic regression was applied with stepwise backward selection. Initially, all factors presenting with *p* < 0.5 in the univariate analysis were included in the model. Then factors that showed no or limited statistically significant association (*p* > 0.1) with overall complication, major complication and non‐home discharge adjusted for the remaining factors in the model were deleted from the model in stepwise fashion. The 10 tested variables for overall and major complication were as follows: age, sex (male), Group A disease, dementia, ASA‐PS, SIRS, operation time, blood lost, laparoscopic procedure, a need for daily care or support.

Abbreviations: ASA‐PS, American association of anesthesia‐physical status; 95% CI, 95% confidence interval; SIRS, systemic inflammatory response syndrome.

^a^ Based on likelihood test adjusted for the other factors in the final model.

^b^ Strangulated bowel obstruction, Upper/Lower bowel perforation and Gastrointestinal ischemia or bleeding.

## 4 Discussion

In this retrospective analysis of emergency surgery for acute abdominal disease in super‐elderly patients aged ≥ 85 years, major complications occurred in 19.4% of cases; however, 72.1% of patients were discharged home. These findings suggest that emergency surgery can be performed safely in this population with acceptable short‐term outcomes. As expected, patients with major complications tended to have prolonged hospitalization and higher inpatient costs. By contrast, even among patients without major complications, inpatient costs were disproportionately high relative to LOS, indicating potential room for cost optimization.

Disease‐specific analysis showed that mortality was concentrated in Group A diseases, which were also associated with higher ICU admission rates. Because several deaths occurred within a few days after surgery (Figure 2), these conditions should be regarded as life‐threatening. Nevertheless, there were no significant differences between Groups A and B in overall complications, major complications, or non‐home discharge. These results indicate that with appropriate intervention, survival and discharge are achievable even in high‐risk conditions, and that intensive allocation of medical resources in such cases is justified. Major complications occurred across disease categories and were significantly dependent on physiological reserve and surgical invasiveness. The results of subgroup analyses suggest that both surgical invasiveness and the presence of abdominal contamination substantially influence postoperative outcomes and resource utilization, indicating that incorporating procedure‐related factors alongside disease pathology may improve risk stratification and cost prediction. Compared with patients without major complications, total inpatient costs were approximately doubled, and fee‐for‐service components increased by nearly threefold. Given the inherent frailty and limited physiological reserve of these patients, such elevated perioperative risk and cost burden should be regarded as an acceptable and unavoidable consequence [11].

Although Japan's DPC system is designed to standardize care quality and promote efficient resource allocation, evidence regarding short‐term surgical outcomes and cost structure for emergency abdominal surgery remains limited. Cost increases associated with major complications and prolonged hospitalization are unavoidable and should not be targets for cost reduction. However, a subset of patients without significant complications experienced prolonged hospital stays and high medical costs. In many of these cases, delayed recovery of oral intake or insufficient rehabilitation hindered timely discharge despite clinical stability. Unnecessary prolongation of LOS may lead to declines in activities of daily living and cognitive function, ultimately reducing the likelihood of discharge to home.

Residual cost analysis demonstrated that excess total inpatient costs and fee‐for‐service components were independently associated with ASA‐PS, disease severity, and surgical invasiveness, but not with age or preoperative care dependency. These findings suggest that surgical decision‐making should not be based on chronological age alone [12, 13]. Instead, functional status, frailty, and social background should be considered when evaluating surgical indications in super‐elderly patients. Functional decline after surgery is almost inevitable in patients aged ≥ 85 years old, therefore, implementing an ERAS protocol—involving early mobilization, early resumption of oral intake, multimodal analgesia, respiratory physiotherapy, and early removal of catheter is also feasible in this population. Furthermore, additional multivariable analysis to compare short stay group and long stay group suggests that prolonged hospitalization in patients without major complications represents a distinct clinical state that cannot be adequately explained by baseline patient characteristics or initial disease severity. Therefore, preventing unnecessary prolongation of hospitalization is of critical importance. Even in the absence of major complications, targeted perioperative interventions for patients with these risk factors—such as intensified postoperative rehabilitation, nutritional support, and early discharge planning—may improve efficiency and facilitate earlier discharge [14]. Delayed resumption of oral intake is a significant contributor to prolonged hospitalization; thus, we place particular emphasis on swallowing rehabilitation, and this effort may positively influence both patient activities of daily living (ADL) and fee‐for‐service costs.

There are several limitations to this study. First, this was retrospective and subject to constraints in data extraction. Therefore, we were unable to compare patients aged ≥ 85 years with younger patients, or to compare clinical and cost outcomes between surgical and non‐surgical groups within each age category. In addition, more detailed information on geriatric vulnerability factors is required to evaluate the validity of surgical indication. Selection bias is thus unavoidable, and the true benefit of aggressive surgical management in the super‐elderly population remains uncertain. Our findings should be interpreted as applying specifically to patients selected for emergency surgery rather than to the entire population of super‐elderly patients with acute abdominal disease. Prospective observational studies would be better suited to address these issues. Second, the specific components responsible for these excessive medical costs—such as rehabilitation, imaging, or laboratory testing—cannot be identified in this retrospective analysis and warrant further investigation. Third, because the DPC system is unique to Japan, the generalizability of these findings to other healthcare systems may be limited. Furthermore, claim points may not directly reflect actual hospital costs.

In conclusion, emergency surgery for acute abdominal disease in super‐elderly patients can be performed safely with appropriate patient selection and can achieve discharge to home in most cases. While medical resources should be appropriately concentrated on high‐risk diseases and patients with major complications, inpatient costs among patients without significant complications may be modifiable through improved perioperative management and more efficient resource utilization.

## Author Contributions

**Masakazu Tatsuno:** data curation. **Yumi Shibuya:** data curation. **Yuta Kobayashi:** conceptualization, methodology, data curation, formal analysis, validation, investigation, visualization, writing – original draft, writing – review and editing, project administration, supervision. **Hirokazu Yamaguchi:** data curation. **Atsuya Kamiyama:** data curation. **Shojiro Hata:** data curation. **Tsuyoshi Ozawa:** data curation. **Keiichiro Hara:** data curation. **Ryo Oikawa:** data curation.

## Funding

The authors have nothing to report.

## Ethics Statement

Approved by the Ethics Committee of Showa General Hospital (No. REC‐395).

## Consent

IC was waived due to the retrospective nature of the study.

## Conflicts of Interest

The authors declare no conflicts of interest.

## Supporting information


**Figure S1:** Patient selection flowchart. Flow diagram of patients admitted for acute abdominal disease. Among patients aged ≥ 85 years, those undergoing emergency surgery were included in the final cohort (*N* = 247).

**Table S1A:** Subgroup and cost analyses, disease category comparison.
**Table S1B:** Subgroup and cost analyses, bowel procedure type comparison.
**Table S1C:** Subgroup and cost analyses, comparison of contaminated infectious conditions and intestinal ischemia/bleeding.
**Table S1D:** Subgroup and cost analyses, comparison among complications.
**Table S2:** Cost component analysis comparing excess and non‐excess groups without major postoperative complications.
**Table S3:** Multivariable analysis of factors associated with excess hospitalization cost for comparing short‐ and long‐stay.

## Data Availability

The data that support the findings of this study are available on request from the corresponding author. The data are not publicly available due to privacy or ethical restrictions.

## References

[1] C. A. Kennedy , D. Shipway , and K. Barry , “Frailty and Emergency Abdominal Surgery: A Systematic Review and Meta‐Analysis,” Surgeon 20 (2022): e307–e314. 34980559 10.1016/j.surge.2021.11.009

[2] E. St‐Louis , M. Sudarshan , M. Al‐Habboubi , et al., “The Outcomes of the Elderly in Acute Care General Surgery,” European Journal of Trauma and Emergency Surgery 42 (2016): 107–113. 26038035 10.1007/s00068-015-0517-9

[3] E. J. Kaufman , C. J. Wirtalla , L. J. Keele , et al., “Costs of Care for Operative and Nonoperative Management of Emergency General Surgery Conditions,” Annals of Surgery 279 (2024): 684–691. 37855681 10.1097/SLA.0000000000006134 PMC10939968

[4] Y. Kobayashi , R. Oikawa , Y. Shibuya , et al., “Validation of the Safe Application of Tokyo Guideline 2018 (TG18) Severity Grading and Treatment Algorithm in Super‐Elderly Patients Aged 85 and Over With Acute Cholecystitis: A Study in the Japanese Tertiary Care Hospital,” Annals of Gastroenterological Surgery 10, no. 2 (2025): 570–577, 10.1002/ags3.70105. 41799575 PMC12962015

[5] A. Murata , K. Okamoto , S. Matsuda , et al., “Multivariate Analysis of Factors Influencing Length of Hospitalization and Medical Costs of Cholecystectomy for Acute Cholecystitis in Japan: A National Database Analysis,” Keio Journal of Medicine 62 (2013): 83–94. 23912168 10.2302/kjm.2012-0015-oa

[6] A. Murata , K. Okamoto , T. Mayumi , K. Maramatsu , and S. Matsuda , “Age‐Related Differences in Outcomes and Etiologies of Acute Abdominal Pain Based on a National Administrative Database,” Tohoku Journal of Experimental Medicine 233 (2014): 9–15. 24739505 10.1620/tjem.233.9

[7] E. Teisberg , S. Wallace , and S. O'Hara , “Defining and Implementing Value‐Based Health Care: A Strategic Framework,” Academic Medicine 95 (2020): 682–685. 31833857 10.1097/ACM.0000000000003122 PMC7185050

[8] K. Okamoto , K. Suzuki , T. Takada , et al., “Tokyo Guidelines 2018: Flowchart for the Management of Acute Cholecystitis,” Journal of Hepato‐Biliary‐Pancreatic Sciences 25 (2018): 55–72. 29045062 10.1002/jhbp.516

[9] P. A. Clavien , J. Barkun , M. L. de Oliveira , et al., “The Clavien‐Dindo Classification of Surgical Complications: Five‐Year Experience,” Annals of Surgery 250 (2009): 187–196. 19638912 10.1097/SLA.0b013e3181b13ca2

[10] K. Hayashida , G. Murakami , S. Matsuda , and K. Fushimi , “History and Profile of Diagnosis Procedure Combination (DPC): Development of a Real Data Collection System for Acute Inpatient Care in Japan,” Journal of Epidemiology 31 (2021): 1–11. 33012777 10.2188/jea.JE20200288 PMC7738645

[11] J. Straatman , M. A. Cuesta , E. S. de Lange‐de Klerk , et al., “Hospital Cost‐Analysis of Complications After Major Abdominal Surgery,” Digestive Surgery 32 (2015): 150–156. 25791798 10.1159/000371861

[12] Z. Cooper , J. W. Scott , R. A. Rosenthal , and S. L. Mitchell , “Emergency Major Abdominal Surgical Procedures in Older Adults: A Systematic Review of Mortality and Functional Outcomes,” Journal of the American Geriatrics Society 63 (2015): 2563–2571. 26592523 10.1111/jgs.13818 PMC4827160

[13] K. Omichi , K. Hasegawa , H. Kumamaru , et al., “Association Between Age and Short‐Term Outcomes of Gastroenterological Surgeries in Older Patients: An Analysis Using the National Clinical Database in Japan,” Langenbeck's Archives of Surgery 406 (2021): 2827–2836. 10.1007/s00423-021-02296-5 34379197

[14] P. Bisagni , V. D'Abrosca , V. Tripodi , et al., “Cost Saving in Implementing ERAS Protocol in Emergency Abdominal Surgery,” BMC Surgery 24 (2024): 70. 38389067 10.1186/s12893-024-02345-y PMC10885507

