# Supplementary material for: Short‐Term Outcomes and Cost Drivers of Emergency Surgery for Acute Abdominal Disease in Super‐Elderly Patients: A Study in the Japanese Tertiary Care Hospital
Source: Ann Gastroenterol Surg. 2026 Apr 10;10(5):1486–95. doi: 10.1002/ags3.70222 (PMC13394618; doi:10.1002/ags3.70222)
Supplement: Supplementary file 1 [file AGS3-10-1486-s001.docx]

| **Supplementary Table S1A.**  **Subgroup and cost analyses, Disease category comparison** | | | |
| --- | --- | --- | --- |
|  | Group A^a^  N=136 | Group B^b^  N=111 | *p* |
| age  sex (male/female)  ASA-PS (1/2/3/4/5)  BMI, kg/m^2^  SIRS score  Laparoscopic procedure  Operation time, min  Estimated blood loss, ml  Transfusion  ICU admission  Overall complication  Major complication  Grade3a/ 3b  Grade4a/ 4b  Mortality  Postoperative length of stay, days  Nonhome discharge  Total inpatient claim points  Fee-for-service component | 87(85-98)  53/ 83  0/77/43/15/1  19.2(13.3-29.1)  1(0-4)  4(2.9)  92(27-297)  25(0-4195)  14(10.3)  18(13.2)  68(50.0)  30(22.1)  5(3.7)/ 0  9(6.6)/ 5(3.7)  11(8.1)  13(2-76)  44（32.4）  137400(24466-610964)  26514(2607-248752) | 87(85-99)  68/ 43  1/58/45/6/1  21.1(13.3-33.8)  1(0-4)  14(12.6)  109(15-335)  60(0-4710)  7(6.3)  4(3.6)  41(36.9)  18(16.2)  9(8.1)/ 2(1.8)  5(4.5)/ 1(0.9)  1(0.9)  10(1-72)  25（22.5）  117838(46993-513713)  22796(1511-155122) | 0.953  <0.001  0.277  0.001  0.035  0.006  0.060  0.005  0.360  0.012  0.053  0.263  0.014  0.032  0.090  0.071  0.236 |
| Abbreviation: ASA-PS; American Society of Anesthesiologists physical status, SIRS; systemic inflammatory response syndrome, ICU; intensive care unit  ^a^ Strangulated bowel obstruction, Upper/ Lower bowel perforation and Gastrointestinal ischemia or bleeding  ^b^ Appendicitis, Cholecystitis, Mechanical bowel obstruction and others | | | |

| **Supplementary Table S1B.**  **Subgroup and cost analyses, Bowel procedure type comparison** | | | | |
| --- | --- | --- | --- | --- |
|  | Bowel surgical procedure type | | |  |
|  | No bowel resection  (N=170) | Resection with anastomosis  (N=49) | Resection with stoma  (N=28) | P value |
| age  sex (male/female)  ASA-PS (1/2/3/4/5)  BMI, kg/m^2^  SIRS score  Disease  Strangulated bowel obstruction  Upper bowel perforation  Lower bowel perforation  Gastrointestinal ischemia or bleeding  Acute appendicitis  Acute cholecystitis  Mechanical bowel obstruction  Others  Laparoscopic procedure  Operation time, min  Estimated blood loss, ml  Transfusion  ICU admission  Overall complication  Major complication  Grade3a/ 3b  Grade4a/ 4b  Mortality  Postoperative length of stay, days  Nonhome discharge  Total inpatient claim points  Fee-for-service component | 87(85-99)  90/ 80  1/ 97/ 63/ 8/ 1  20.3(13.3-33.8)  1(0-4)  64(37.7)  10(5.9)  1(0.6)  0  24(14.1)  48(28.2)  20(11.8)  3(1.8)  18(10.6)  90(15-335)  25(0-4710)  8(4.7)  5(2.9)  65(38.2)  24(14.2)  11(6.5)/2(1.2)  5(2.9)/ 1(0.6)  5(2.9)  10(1-76)  43(25.3)  109407(24466-513713)  19944(1511-248752) | 87(85-98)  19/ 30  0/ 24/ 17/ 7/ 1  20.2(14.2-26.4)  1(0-4)  27(55.1)  0  4(8.2)  8(16.3)  0  0  10(20.4)  0  0  119(42-297)  60(0-4195)  8(16.3)  5(10.2)  24(49.0)  8(16.3)  2(4.1)/ 0  1(2.0)/2(4.1)  3(6.3)  13(2-57)  15(30.6)  147959(72730-507583)  26723(2607-165198) | 88(85-96)  12/ 16  0/ 14/ 8/ 6/ 0  19.2(14.2-29.1)  1(0-4)  0  0  21(75.0)  1(3.6)  0  0  5(17.9)  1(3.6)  0  155(25-282)  65(0-1245)  5(17.9)  12(42.9)  20(71.4)  16(57.1)  1(3.6)/0  8(28.6)/3(10.7)  4(14.3)  23(3-55)  11(39.3)  223752(98748-610964)  47380(7028-246231) | 0.332  0.172  0.109  0.905  0.017  <0.001  0.012  <0.001  0.004  0.006  <0.001  0.004  <0.001  0.032  <0.001  0.279  <0.001  <0.001 |
| Abbreviation: ASA-PS; American Society of Anesthesiologists physical status, BMI; body mass index, SIRS; systemic inflammatory response syndrome, ICU; intensive care unit | | | | |

| **Supplementary Table S1C.**  **Subgroup and cost analyses, comparison of contaminated infectious conditions and intestinal ischemia/bleeding** | | | |
| --- | --- | --- | --- |
|  |  | | |
|  | Contaminated infectious conditions  (Upper/Lower bowel perforation,  Acute appendicitis, Acute cholecystitis, Others)  (N=112) | Intestinal ischemia or bleeding  (Strangulated/ mechanical bowel obstruction,  Gastrointestinal ischemia or bleeding)  (N=135) | P value |
| age  sex (male/female)  ASA-PS (1/2/3/4/5)  BMI, kg/m^2^  SIRS score  Laparoscopic procedure  Operation time, min  Estimated blood loss, ml  Transfusion  ICU admission  Overall complication  Major complication  Grade3a/ 3b  Grade4a/ 4b  Mortality  Postoperative length of stay, days  Nonhome discharge  Total inpatient claim points  Fee-for-service component | 87(85-99)  67/ 45  1/ 61/ 39/ 10/ 1  21.7(14.2-33.8)  1(0-4)  12(10.7)  124(15-282)  90(0-4710)  14(12.5)  14(12.5)  50(44.6)  29(25.9)  8(7.1)/ 2(1.8)  9(8.0)/ 2(1.8)  8(7.1)  11(1-55)  28(25.0)  134779(46993-610964)  27606(1511-246231) | 88(85-99)  54/ 81  0/ 74/ 49/ 11/ 1  19.0(13.3-26.4)  1(0-4)  6(4.4)  82(25-335)  20(0-2387)  7(5.2)  8(5.9)  59(43.7)  19(14.1)  6(4.4)/ 0  5(3.7)/ 4(3.0)  4(3.0)  11(2-76)  41(30.4)  123243(24466-513713)  21724(4103-248752) | 0.211  0.002  0.860  <0.001  <0.001  0.084  <0.001  <0.001  0.065  0.077  0.898  0.024  0.149  0.980  0.394  0.037  0.076 |
| Abbreviation: ASA-PS; American Society of Anesthesiologists physical status, BMI; body mass index, SIRS; systemic inflammatory response syndrome, ICU; intensive care unit | | | |

| **Supplementary Table S1D.**  **Subgroup and cost analyses, comparison among complications** | | | | |
| --- | --- | --- | --- | --- |
|  | Pneumoniae  (N=16) | Intra-abdominal abscess  (N=6) | Anastomotic leakage  (N=2) | P value |
| Postoperative length of stay, days  Nonhome discharge  Total inpatient claim points  Fee-for-service component | 17(2-47)  9(56.3)  184248(77202-610964)  39094(12771-246231) | 25(13-48)  1(16.7)  182393(104423-292825)  30674(15174-87144) | 65(57-72)  2(100)  479442(451301-507583)  107200(86892-127507) | 0.034  0.086  0.112  0.188 |
|  | | | | |

| **Supplementary Table 2.** Cost component analysis comparing excess and non-excess groups without major postoperative complications | | | |
| --- | --- | --- | --- |
|  | Excess cost group  (N=49) | Non-excess group  (N=150) | P　value |
| Total inpatient claim points  Surgical procedure points  Anesthesia + Operation room consumables  DPC bundled component  Fee-for-service component | 159218(24466-273635)  31226(8880-87789)  15215(9711-28044)  59735(22363-132016)  47207(24466-103667) | 105011(36208-315106)  26805(6740-116896)  14598(8611-28466)  44530(9378-187963)  17307(1511-62190) | <0.001  0.080  0.187  0.008  <0.001 |
| Abbreviation: DPC; Diagnosis Procedure Combination | | | |

| **Supplementary Table3.**  **Multivariable analysis of factors associated with excess hospitalization cost for comparing short- and long-stay** | | | | | | | | |
| --- | --- | --- | --- | --- | --- | --- | --- | --- |
| **Short stay group (N=102): Length of stay ≤10 days ^b^** | | | |  | **Long stay group (N=97) Length of stay ≥11 days ^b^** | | | |
|  | p value ^a^ | Odds Ratio | 95%CI |  |  | p value ^a^ | Odds Ratio | 95%CI |
| **The excess cost for quartile of Total inpatient fee point (the top 25%)** | | | | | | | | |
| Sex (male) | 0.043 | 4.22 | 1.05-16.9 |  | Operation time (+1 hour) | ＜0.001 | 5.91 | 2.62-13.3 |
| ASA-PS (+1) | 0.021 | 3.40 | 1.21-9.57 |  |  |  |  |  |
| SIRS score (+1) | 0.003 | 3.12 | 1.47-6.60 |  |  |  |  |  |
| Operation time (+1 hour) | 0.013 | 3.71 | 1.31-10.5 |  |  |  |  |  |
| **The excess cost for quartile of fee-for-service component (the top 25%)** | | | | | | | | |
| Sex (male) | 0.017 | 3.94 | 1.28-12.2 |  | Operation time (+1 hour) | 0.001 | 2.53 | 1.44-4.46 |
| a: a Based on likelihood test adjusted for the other factors in the final model  b: Using the median length of stay (LOS) as the cutoff, patients were divided into two groups  Abbreviation: ASA-PS, American association of anesthesia-physical status; 95%CI, 95% confidence interval; SIRS, systemic inflammatory response syndrome  Multivariate logistic regression was applied with stepwise backward selection. Initially, all factors presenting with P < 0.5 in the univariate analysis were included in the model. Then factors that showed no or limited statistically significant association (P > 0.1) with overall complication, major complication and non-home discharge adjusted for the remaining factors in the model were deleted from the model in stepwise fashion.  The 10 tested variables for overall and major complication were as follows:  age, sex(male), GroupA disease, dementia, ASA-PS, SIRS, operation time, blood lost, laparoscopic procedure, a need for daily care or support | | | | | | | | |
