# Supplementary figures and images for: Short‐Term Outcomes and Cost Drivers of Emergency Surgery for Acute Abdominal Disease in Super‐Elderly Patients: A Study in the Japanese Tertiary Care Hospital
Source: Ann Gastroenterol Surg. 2026 Apr 10:10.1002/ags3.70222. Online ahead of print. doi: 10.1002/ags3.70222 (PMC13394618; doi:10.1002/ags3.70222)

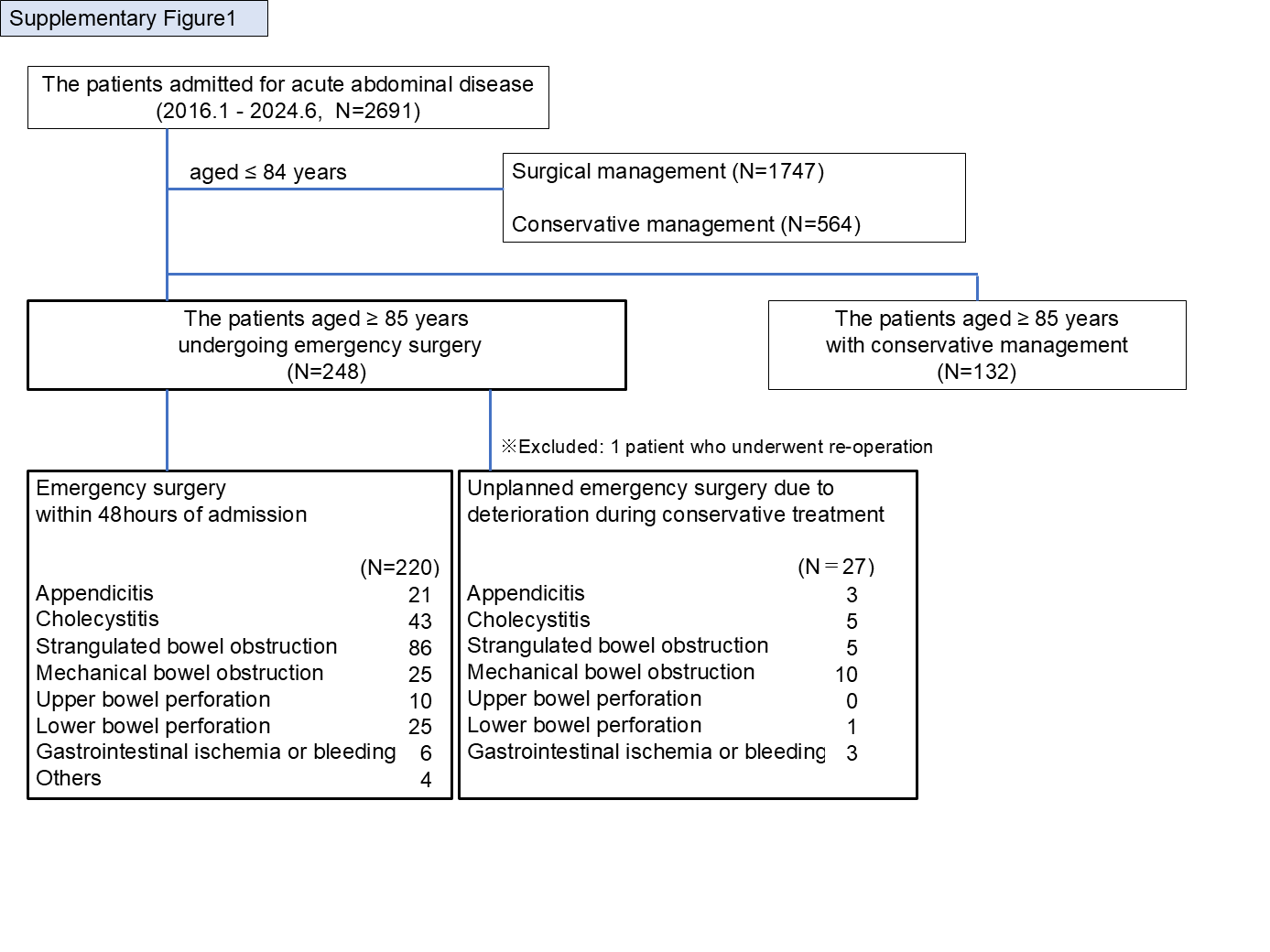

Supplement: Supplementary file 1 — Figure S1: Patient selection flowchart. Flow diagram of patients admitted for acute abdominal disease. Among patients aged ≥ 85 years, those undergoing emergency surgery were included in the final cohort (N = 247). [file AGS3-9999-0-s002.tif]
